# Hybrid Model for Clinical Post-Graduate Teaching in a University Hospital

**DOI:** 10.5704/MOJ.2011.035

**Published:** 2020-11

**Authors:** SA Shamsul, T Cheng, AA Abbas

**Affiliations:** Department of Orthopaedic Surgery, University of Malaya, Kuala Lumpur, Malaysia

Dear Editor,

We read with interest the article by Tay *et al* “COVID-19 in Singapore and Malaysia: Rising to the Challenges of Orthopaedic Practice in an Evolving Pandemic”, published in the last issue of the Malaysian Orthopaedic Journal^1^. We write in to share our own experience.

COVID-19 caught the world by surprise and has shaken us out of our comfort zones, teaching us to accept and practice many “new normals”. One of these would be “social distancing”, which means to put a physical distance (usually >1m) between individuals.

Following the Movement Control Order (MCO) in March 2020, all group related activities were stopped and teaching sessions were moved to online platforms; such as Zoom, Google Meet and Microsoft Teams. Two to three presenters were scheduled weekly to present on topics according to the different subspecialties.

As the COVID-19 situation in Malaysia improved in June, we were allowed to restart group sessions while adhering to strict protocols. Previously, clinical teaching sessions for post-graduate Orthopaedic trainees were held twice a week in a conference room that is usually packed with about 50 people. We had to figure out a way to limit attendees while providing an avenue for other trainees to participate without physically gathering together.

We designed a *“Hybrid Clinical Teaching Model”* which incorporated full audio-video streaming over the internet with limited number of live participants in a conference room.

Patients, trainees and lecturers were all given portable microphones while a camera was placed strategically to capture relevant clinical signs and tests. This enabled other trainees to participate in the session without needing to be physically present. Attendance was limited to post-graduate trainees and faculty members only, and recording was prohibited to protect patients’ privacy.

The Microsoft Teams© platform was chosen as it is subscribed by the university and all faculty members and trainees are identifiable via their usernames.

**Fig. 1:**
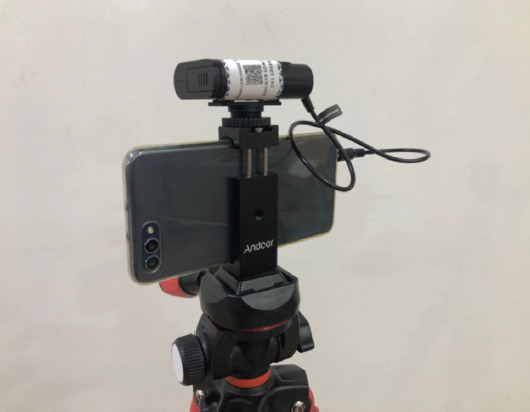
Android smartphone connected to wireless microphone receiver mounted on a tripod.

**Fig. 2:**
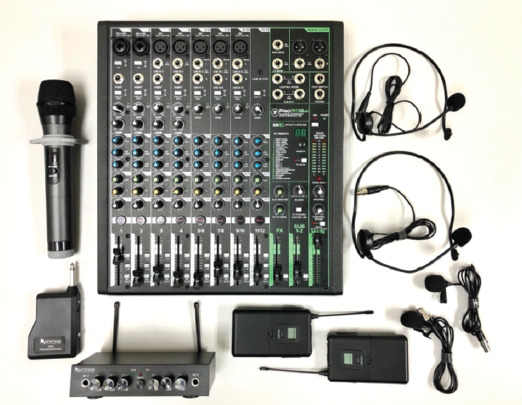
12-channel USB Mixer with various wireless microphones and receiver for audio input.

A smartphone connected to wireless microphones (two wireless microphones with one receiver) mounted on a tripod was used (Fig. 1) for both audio-video streaming, while additional microphones were used for question and answer discussions between participants and faculty members when needed. Additional microphones were streamed online via a single sound mixer (Fig. 2) to improve clarity and reduce lag. Feedback has been encouraging thus far, with some online participants stating that the experience is more immersive than sitting at the last row in the actual conference room.

**Fig. 3:**
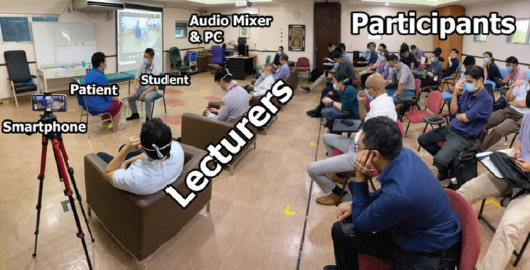
Layout in the conference room consisting of lecturers, students, live patient, smartphone and audio setup.

This setup enabled us to have 50-60 participants every session, 15 physically in the room while others joined online via Microsoft Teams (Fig. 3). Our trainees who were off-campus on attachment at other centres were also able to attend the online sessions. This “Hybrid Clinical Teaching Model” not only allows us to adhere to distancing protocols but has also increased the reach of our teaching sessions. That which seemed to be an inconvenience at first glance has now become a powerful tool in the training of orthopaedic surgeons.
